# Kinetics and persistence of cellular and humoral immune responses to SARS‐CoV‐2 vaccine in healthcare workers with or without prior COVID‐19

**DOI:** 10.1111/jcmm.17186

**Published:** 2022-01-18

**Authors:** Mihaela Chivu‐Economescu, Coralia Bleotu, Camelia Grancea, Daniela Chiriac, Anca Botezatu, Iulia V. Iancu, Ioana Pitica, Laura G. Necula, Ana Neagu, Lilia Matei, Denisa Dragu, Camelia Sultana, Elena L. Radu, Alina Nastasie, Oana Voicu, Marius Ataman, Saviana Nedeianu, Cristina Mambet, Carmen C. Diaconu, Simona Maria Ruta

**Affiliations:** ^1^ Stefan S. Nicolau Institute of Virology Bucharest Romania; ^2^ Carol Davila University of Medicine and Pharmacy Bucharest Romania; ^3^ Institute for Water Quality and Resource Management TU Wien Vienna Austria

**Keywords:** antibody levels, IFN**‐**γ, immune response post‐infection, immune response post‐vaccination, mRNA vaccine, neutralizing antibodies, SARS‐CoV‐2 infection, spike‐specific CD4^+^ T cell, spike‐specific CD8^+^ T cell

## Abstract

SARS‐CoV‐2 vaccines are highly efficient against severe forms of the disease, hospitalization and death. Nevertheless, insufficient protection against several circulating viral variants might suggest waning immunity and the need for an additional vaccine dose. We conducted a longitudinal study on the kinetics and persistence of immune responses in healthcare workers vaccinated with two doses of BNT162b2 mRNA vaccine with or without prior SARS‐CoV‐2 infection. No new infections were diagnosed during follow‐up. At 6 months, post‐vaccination or post‐infection, despite a downward trend in the level of anti‐S IgG antibodies, the neutralizing activity does not decrease significantly, remaining higher than 75% (85.14% for subjects with natural infection, 88.82% for vaccinated after prior infection and 78.37% for vaccinated only). In a live‐virus neutralization assay, the highest neutralization titres were present at baseline and at 6 months follow‐up in persons vaccinated after prior infection. Anti‐S IgA levels showed a significant descending trend in vaccinated subjects (*p* < 0.05) after 14 weeks. Cellular immune responses are present even in vaccinated participants with declining antibody levels (index ratio 1.1–3) or low neutralizing activity (30%–40%) at 6 months, although with lower T‐cell stimulation index (*p* = 0.046) and IFN**‐**γ secretion (*p* = 0.0007) compared to those with preserved humoral responses.

## 1 INTRODUCTION

Severe acute respiratory syndrome coronavirus‐2 (SARS‐CoV‐2) spread rapidly around the globe infecting by the end of November 2021 over 250 million individuals and causing over 5 million deaths. In December 2020, the first vaccines became available, and until the end of November 2021, more than 8 billion doses were administered worldwide, with about 55% of the world population currently immunized.^1^

Immunity gained from natural infection or vaccination provides a significant degree of protection against both reinfection and progression towards a severe form of COVID‐19 requiring hospitalization. It is currently estimated that 89% of the individuals recovered after a natural infection are protected for at least 8 months,^2^ while vaccinated people have a reduction of 50%–95% in their risk of infection,^3^ depending on the type of vaccine, the interval between doses, time elapsed from the completion of the vaccination regimen, age, immuno‐competence, prior infection with SARS‐CoV‐2 and the circulating viral variants. During the clinical trials, mRNA‐based vaccines have demonstrated the highest efficacy against symptomatic COVID‐19: 95% for BNT162b2 (Pfizer/BioNTech)^4^ and 94% for mRNA‐1273 (Moderna),^5^ followed by vector‐based vaccines: 92% efficacy against symptomatic infection for Gam‐COVID‐Vac (Gamaleya),^6^ 70% for ChAdOx1‐nCoV19 (AstraZeneca)^7^ and 67% for Ad26.COV2.S (Janssen).^8^

After introducing large‐scale vaccination, numerous independent studies confirmed the real‐world effectiveness of vaccination, with results similar to the clinical trials during the dominance of Alpha variant and a slight decrease, especially against symptomatic infection, after emergence and global spread of Delta variant.

Currently, there is a debate on the necessity of additional vaccine doses, with supporters citing waning immunity and decreased efficacy against viral variants and opponents highlighting incomplete scientific evidence, differences according to the individual immuno‐competence status, high risk of exposure, as well as ethical reasons related to inequalities in vaccine access. In August 2021, the US CDC published a report showing that mRNA vaccine protection against infection has declined from 91.7% in May to 79.8% in July, while vaccine effectiveness against hospitalization remained relatively stable (91.9%–95.3%).^9^ Another CDC study showed a more pronounced decline of mRNA vaccines efficiency against infection among nursing home residents (from 74.7% in March 2021 to 53.1% in July 2021, when B.1.617.2 (Delta) variant became dominant).^10^

In the general population, the number of severe SARS‐CoV‐2 infections after vaccination remains low, with more than 40% of hospitalized breakthrough cases registered in immunocompromised persons,^11^, ^12^ who are at risk for prolonged SARS‐CoV‐2 shedding and viral evolution. Preliminary unpublished data from Israel suggest a major drop down in vaccine effectiveness against SARS‐CoV‐2 infection (39%), especially in those vaccinated in the early months of 2021, with protection retained against hospitalization (81%).^13^ Similar data were reported in a test negative, case‐control study in Qatar with significantly diminished levels of protection against symptomatic and asymptomatic infection 20 weeks after the second dose of BNT162b2 vaccine, but maintenance of protection against severe, critical and fatal forms of COVID‐19.^14^

Furthermore, two studies reported by Public Health England showed only modest differences in effectiveness against symptomatic infection caused by Alpha and Delta variants in subjects who received either two doses of BNT162b2 mRNA vaccine: (93.7% for Alpha variant and 88% for Delta variant) or ChAdOx1‐nCoV‐19 vaccine, (74.5% against Alpha variant and 67% against Delta variant).^15^

Israel was the first country to recommend an additional dose of mRNA vaccine for all persons over 30 years, communicating encouraging results with increased protection against the highly transmissible Delta variant 1 week after the booster dose (Israeli HMO Maccabi unpublished data). The United States and several European countries have approved the third dose of mRNA vaccines for immunosuppressed persons, based on the lower efficacy of a two doses vaccine regimen and on emerging evidence of seroconversion or increased levels of neutralizing antibodies after additional vaccine doses.^16^

In order to make additional public health decisions related to the need for an extended vaccination regimen, additional data on the duration of natural and vaccine‐induced immune responses are needed. In this regard, we conducted a pilot longitudinal study on the kinetics and persistence of immune responses in subjects vaccinated with 2 doses of BNT162b2 mRNA vaccine or with prior SARS‐CoV‐2 infection. The study assessed the 6 months humoral and cellular immune responses of healthcare workers in a diagnostic and research institute in Bucharest, Romania. As of August 2021, more than 5 million persons have been vaccinated in Romania (31% of the population), out of which almost 4 million received Pfizer‐BioNTech BNT162b2 mRNA vaccine.

## 2 METHODS

### 2.1 Enrolment and sample collection

Healthcare personnel (*n* = 56) from Stefan S. Nicolau Institute of Virology, Bucharest, were enrolled to this study in January 2021 and observed longitudinally until August 2021. The institutional review board approved the study, and all participants signed informed consent prior to enrolment.

According to their baseline status, 35 participants were SARS‐CoV‐2 naive (mean age 46.62 ± 13.29 years), while 21 participants had a prior SARS‐CoV‐2 infection (mean age 48.43 ± 12.10 years). From all participants, 71.43% (*N* = 40) accepted vaccination with an mRNA‐based vaccine (Pfizer‐BioNTech BNT162b2; two doses at 21 days interval), the percentage being higher in those without prior infection: 88.57% (31/35), vs. 42.85% (9/21) in those with prior SARS‐CoV‐2 infection. There were no significant age or comorbidities differences between the two groups (*p* > 0.05).

**FIGURE 1:**
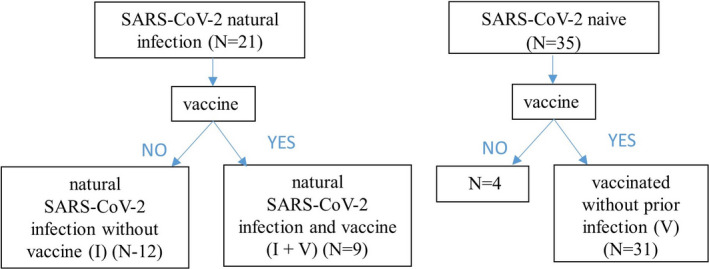
Schematic representation of the groups investigated for the longitudinal immune response

Based on these data, we divided the participants as follows: subjects having had (i) SARS‐CoV‐2 infection and no vaccine (I) (*N* = 12), (ii) prior SARS‐CoV‐2 infection and vaccinated (I + V) (*N* = 9) and (iii) vaccinated without evidence of prior infection (V) (*N* = 31) (Figure 1).

We assessed the humoral and cellular responses longitudinally during 6 months: T1 at 1 week after the second dose of vaccine, T2 at 6 weeks, T3 at 14 weeks and T4 at 26 weeks (W). For participants with SARS‐CoV‐2 infection without vaccine (I), we set up similar evaluation points: T1, T1 + 6W, T1 + 14W and T1 + 26W, where T1 is time from the estimated moment of infection to the first sampling time. Average T1 was 10.85 weeks (3.8–37.14).

### 2.2 Sample processing

10 ml of venous blood was collected on EDTAK3, centrifuged at 1500 *g* for 15 min to separate plasma that was stored at −80°C for downstream antibody analysis.

For cellular immunity analysis, peripheral blood mononuclear cells (PBMC) were separated onto Ficoll‐Paque PLUS density gradient media (1.077 g/ml), washed with RPMI and treated with ACK erythrocyte lysis buffer (Thermo Fisher Scientific) for 5 min. Cells were washed again and used in ex vivo stimulation experiments.

### 2.3 SARS‐CoV‐2 anti‐spike (S) IgG, IgA and anti‐nucleocapsid (NCP) IgM immunoassays

Anti‐S IgG and IgA isotypes and anti‐NCP IgM antibodies were determined using commercially available enzyme‐linked immunosorbent assays (EUROIMMUN Medizinische Labordiagnostika AG). All immunoassays were used and interpreted according to the manufacturer's instructions. Briefly, 1/100 diluted samples were incubated in the wells coated with recombinant S or NCP protein of SARS‐CoV‐2, specific anti‐S IgG and IgA antibodies and anti‐NCP IgM antibodies were identified in an immuno‐enzymatic reaction. Results are expressed as a ratio calculated as the OD 450 of the subject sample over the OD 450 of a calibrator (an anti‐S, or anti‐NCP positive, human IgG, IgA or IgM provided by the manufacturer). This ratio is interpreted as follows: <0.8 negative; ≥0.8 to <1.0 borderline; ≥1.1 positive.

### 2.4 Surrogate SARS‐CoV‐2 virus neutralization test

In order to assess the antibodies neutralizing capacity, a commercial SARS‐CoV‐2 surrogate virus neutralization test (sVNT) was used (GenScript cPass™ SARS‐CoV‐2 Neutralization Antibody Detection Kit, Genscript). This functional assay is based on antibody‐mediated blockade of the interaction between ACE2 receptor and the receptor‐binding domain (RBD) of the spike protein. The method was performed as previously described.^17^ Briefly, plasma samples, positive and negative controls were diluted 1:10 and mixed with an equal volume of enzyme‐conjugated RBD, incubated at 37°C for 30 min and transferred to a plate coated with recombinant human ACE2 for 15 min. After plate washing, the enzyme substrate (100 µl tetramethylbenzidine) was added for 15 min at room temperature, and the optical density (OD) was assessed at 450 nm. Each sample's neutralizing capacity was calculated using the following formula: % inhibition = (1 − (OD450 of sample/Average OD450 of negative controls) × 100. A value of >30% inhibition is considered positive for neutralizing activity.

### 2.5 Viral isolation and whole‐genome sequencing

The virus was isolated from 500 µl of nasopharyngeal swab incubated on a confluent monolayer of Vero E6 cells for 1 h at 37°C followed by addition of Dulbeco's Minimum Essential Medium (DMEM) supplemented with 2% foetal bovine serum (FBS).

Confirmation of virus identity was done by sequencing with the MiSeq system (Illumina). Libraries were prepared using TruSeq Stranded Total RNA Library Prep Gold kit (Illumina). Data analysis was performed with the Illumina Local Run Manager (LRM) Resequencing Module, The Illumina^®^ DRAGEN COVID Lineage App and further lineage/clade analysis were done using Pangolin and NextClade. The sequence was submitted to NextStrain (https://nextstrain.org/ncov/gisaid/global?tl=author).

### 2.6 In vitro virus neutralization assay

In vitro neutralizing assay was performed using a viral strain isolated from a patient diagnosed with SARS‐CoV‐2 infection and confirmed as an Alpha variant (B.1.408 betacoronavirus hCoV‐19/Romania/IIsolate3247BLVL/2020) by virus whole‐genome sequencing using MiSeq NGS system (Illumina) (GSAID accession ID: EPI_ISL_1081959).

Patients’ plasma samples were decomplemented by heat inactivation (56°C for 1 h), subjected to serial twofold dilution (1:2–1:512) in microtitre plates and incubated with 100 median tissue culture infectious dose (TCID) 50 of SARS‐CoV‐2 in a 1:1 ratio for 1 h at 37°C. 100 µl antibody‐virus mixture was inoculated for 1 h at 37°C, on VERO E6 cells, seeded in 96‐well plates at a concentration of 7500 cells per well the day before. The inoculum was removed, and a maintenance medium with 2% FBS was added. The cytopathic effect (CPE) was quantified using IncuCyte Live Cell Imaging System 5 days after inoculation. Neutralizing antibody titre was determined by identifying the highest plasma dilution without observable CPE and expressed as a geometric mean titre (GMT).^18^

### 2.7 Anti‐SARS‐CoV‐2 specific cellular immunity

Specific cellular responses were assessed using ex vivo stimulation of PBMC with a pool of lyophilized peptides (PepTivator SARS‐CoV‐2 Prot_S, Miltenyi Biotec), consisting of 15‐mer sequences with 11 amino acids overlap, covering the immunodominant sequence domains of the spike glycoprotein of SARS‐CoV‐2. PBMCs were added in 96 wells plates, in a concentration of 1 × 10^6^, in RPMI1640 supplemented with 10% human serum and subsequently stimulated with 1 µg/ml PepTivator. For the positive and negative control, PBMCs were stimulated with 2.5 µg/ml Phytohemagglutinin‐L (Invitrogen, Thermo Scientific) or 2 µl of sterile water with 10% DMSO, respectively. Cells were incubated for 20 h at 37°C, 5% CO_2_. After specific stimulation, the cells were labelled with specific antibodies for flow cytometric analysis of lymphocytes and supernatants were harvested for interferon gamma (IFN**‐γ**) release detection.

### 2.8 Flow cytometry

Cryopreserved cells were thawed, washed and stimulated for flow cytometry determinations using activation‐induced cell marker (AIM) assays. SARS‐CoV‐2‐specific CD4, CD8, B and NK cells were analysed after *ex vivo* stimulation of PBMCs with PepTivator for 20 h using tetraCHROME CD45‐FITC/CD4‐PE/CD8‐ECD/CD3‐PC5 and tetraCHROME CD45‐FITC/CD56‐PE/CD19‐ECD/CD3‐PC5 antibody cocktails (Beckman Coulter Life Sciences). All tests for individual subjects were run in the same experiment to minimize batch effects. Samples were acquired on a Beckman Coulter EPICS XL flow cytometer. Data were analysed using Kaluza Analysis Software (Beckman Coulter Life Sciences).

### 2.9 IFN‐γ detection

Interferon‐gamma release assay was used to determine the magnitude of the SARS‐CoV‐2‐specific T‐cell response. Supernatants from 1 × 10^6^ PBMC stimulated with PepTivator SARS‐CoV‐2 Prot_S (Miltenyi Biotec) for 20 h, were analysed for IFN**‐γ** concentrations by ELISA, according to manufacturer's instructions (R&D Systems).

### 2.10 Statistical analysis

Data were analysed using GraphPad Prism 5 software. Line plots present means with a 95% confidence interval. Antibody responses were compared between groups using 1‐way ANOVA Tukey's multiple comparison test. For correlations between two groups, Pearson *r* correlation coefficient was used. All tests were performed with a nominal significance threshold of *p* < 0.05. *p < 0.05, **p < 0.01 and ***p < 0.001.

## 3 RESULTS

### 3.1 The kinetics of antibody responses

**FIGURE 2:**
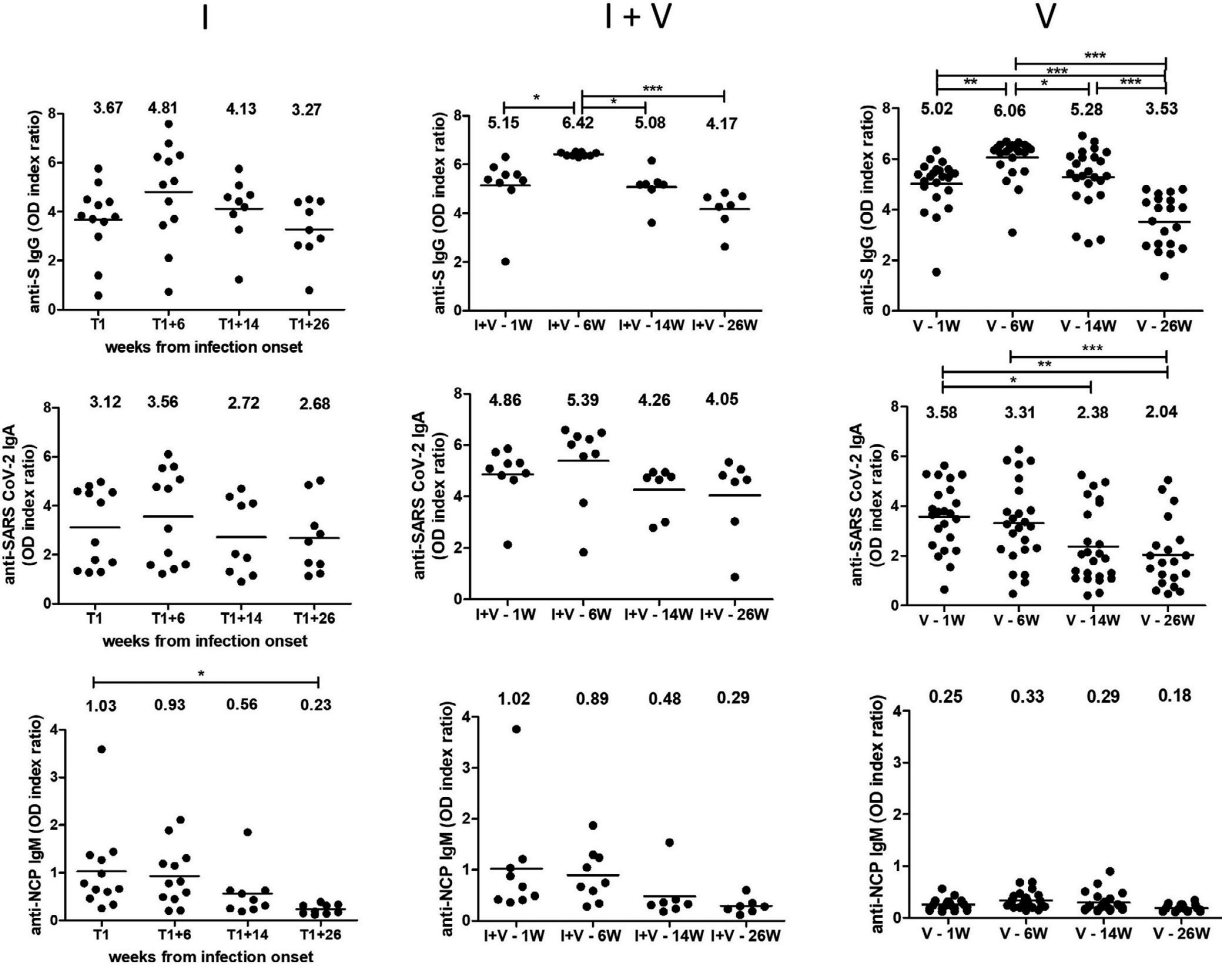
Changes in antibody levels over time. Sequential analysis of isotypic antibody responses (anti‐S IgG, IgA and anti‐NCP IgM) to SARS‐CoV‐2 for plasma samples collected over a 6 months period. Groups description: natural SARS‐CoV‐2 infection without vaccine (I), with prior SARS‐CoV‐2 infection and vaccine (I + V), and vaccinated without prior infection (V). *p < 0.05, **p < 0.01 and ***p < 0.001

The 6 months longitudinal profiles of specific antibodies (anti‐Spike IgG, anti‐Spike IgA, anti‐NCP IgM) and their neutralizing capacity in non‐vaccinated subjects with prior infection (I) versus vaccinated only (V) and vaccinated with prior infection (I + V) participants are presented in Figure 2. The vaccinated subjects were investigated at 4 time points (1, 6, 14 and 26 Ws) after administration of the second vaccine dose while participants with natural SARS‐CoV‐2 infection without vaccine (I) were sampled at T1, T1 + 6W, T1 + 14W and T1 + 26W (average T1 was 10.85 Ws).

There was no evidence of new infections in the enrolled subjects (no cases with positive anti‐NCP IgM antibodies, no symptomatic infections).

All vaccinated subjects, irrespective of their prior infection status, developed anti‐spike IgG and IgA antibodies at 1 week after the second dose of vaccine, with only one slow seroconvertor with detectable antibodies only after 6 weeks post‐second dose. There were no significant differences in the antibody levels or in their neutralizing capacity according to age, BMI or presence of comorbidities. The highest level of antibodies was found in vaccinated subjects with prior SARS‐CoV‐2 infection (I + V group). Anti‐S IgG levels peaked at 6 weeks post‐vaccination (reactivity 6.42 ± 0.07 for I + V and 6.06 ± 0.80 for V group), followed by a slight, but statistically significant decrease (4.17 ± 0.76, *p* = 0.045) for I + V group and a more sharp decrease (3.52 ± 1.03, *p* < 0.0001) for V group.

In SARS‐CoV‐2‐infected subjects, the anti‐S IgG antibodies peaked at 18–20 weeks after infection onset (4.81 ± 2.02) and decreased slowly after 38 weeks (3.27 ± 1.21). One subject infected during the early pandemic period maintained high levels of neutralizing antibodies for 16 months post‐infection.

Vaccination elicits the highest levels of anti‐S IgA antibodies in persons who had prior infection, while subjects who were only vaccinated or only infected had variable responses. Nevertheless, after 14 weeks, there was a significant descending trend for IgA levels in vaccinated subjects (*p* < 0.05).

### 3.2 Changes in neutralizing antibody capacity over time

**FIGURE 3:**
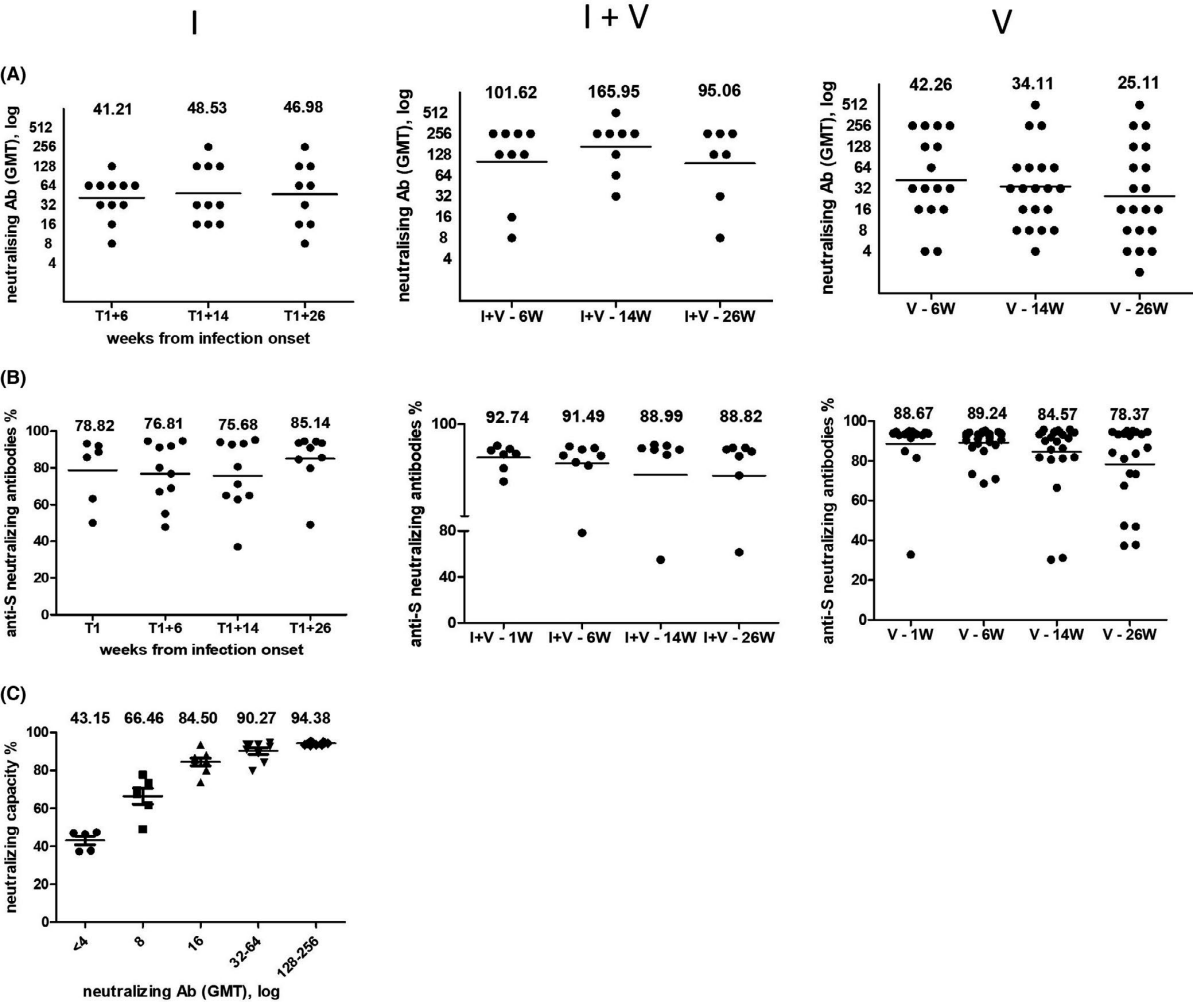
Dynamic of neutralizing antibody using both a classic and a surrogate virus neutralization test. The neutralizing antibody titres and capacity of different groups were compared over time. (A) The titres were measured by whole‐virus replication assay and are expressed as geometrical mean titre (GMT). (B) Results from surrogate virus neutralization test. The neutralizing capacity is expressed as percentage. A cut‐off value ≥30% was used as positive result. (C) Correlations between values obtained in the ELISA surrogate neutralization and the virus neutralization. Groups description: natural SARS‐CoV‐2 infection without vaccine (I), with prior SARS‐CoV‐2 infection and vaccine (I + V), and vaccinated without prior infection (V). Horizontal lines indicate median values

We compared the dynamic of neutralizing antibody using both a classic and a surrogate virus neutralization test. The highest virus neutralization titres were present both at baseline and at 6 months follow‐up in persons vaccinated after a prior infection. Plasma samples from subjects with prior SARS‐CoV‐2 infection exhibited a mean neutralizing antibody titre of 46.98 (95% CI: 19.36–114.28) in unvaccinated individuals vs. 95.06 (95% CI: 28.12–322.10) in vaccinated ones. Unvaccinated subjects with prior SARS‐CoV‐2 infection had lower but stable neutralizing titres. In vaccinated only subjects, the initial virus neutralization titre was similar to that of individuals with the prior infection, but it declined by 40.6% with a significant individual heterogeneity over time (Figure 3A).

In the surrogate functional assay, at 6 weeks post‐infection or post‐vaccine, all plasma samples demonstrated a high neutralizing capacity. Overall, at 26 weeks follow‐up, the median neutralization capacity does not decrease significantly in any of the analysed groups, remaining higher than 75% (85.14% for I, 88.82% for I + V and 78.37% for V). Interestingly, the neutralizing capacity increased by about 10% between months 3 and 6 in people who have gone through infection (I group), while in vaccinated only people (V group), there is a slight decrease, correlated with the decrease in anti‐S IgG antibodies (Figure 3B).

The obtained results showed a correlation between values obtained in the ELISA surrogate neutralization and the virus neutralization (Figure 3C). Thus, titres ≤4 correspond to an average neutralization capacity of 43.15% ± 5.16%, titres of 8 corresponds to 66.46% ± 10.13%, titres of 16 corresponds to 84.50% ± 5.79%, titres of 32–64 correspond to 90.27% ± 5.08% and titres ≥128 to neutralization capacities of over 94.38% ± 0.67%.

### 3.3 Positive correlation between antibody levels and neutralizing capacity

**FIGURE 4:**
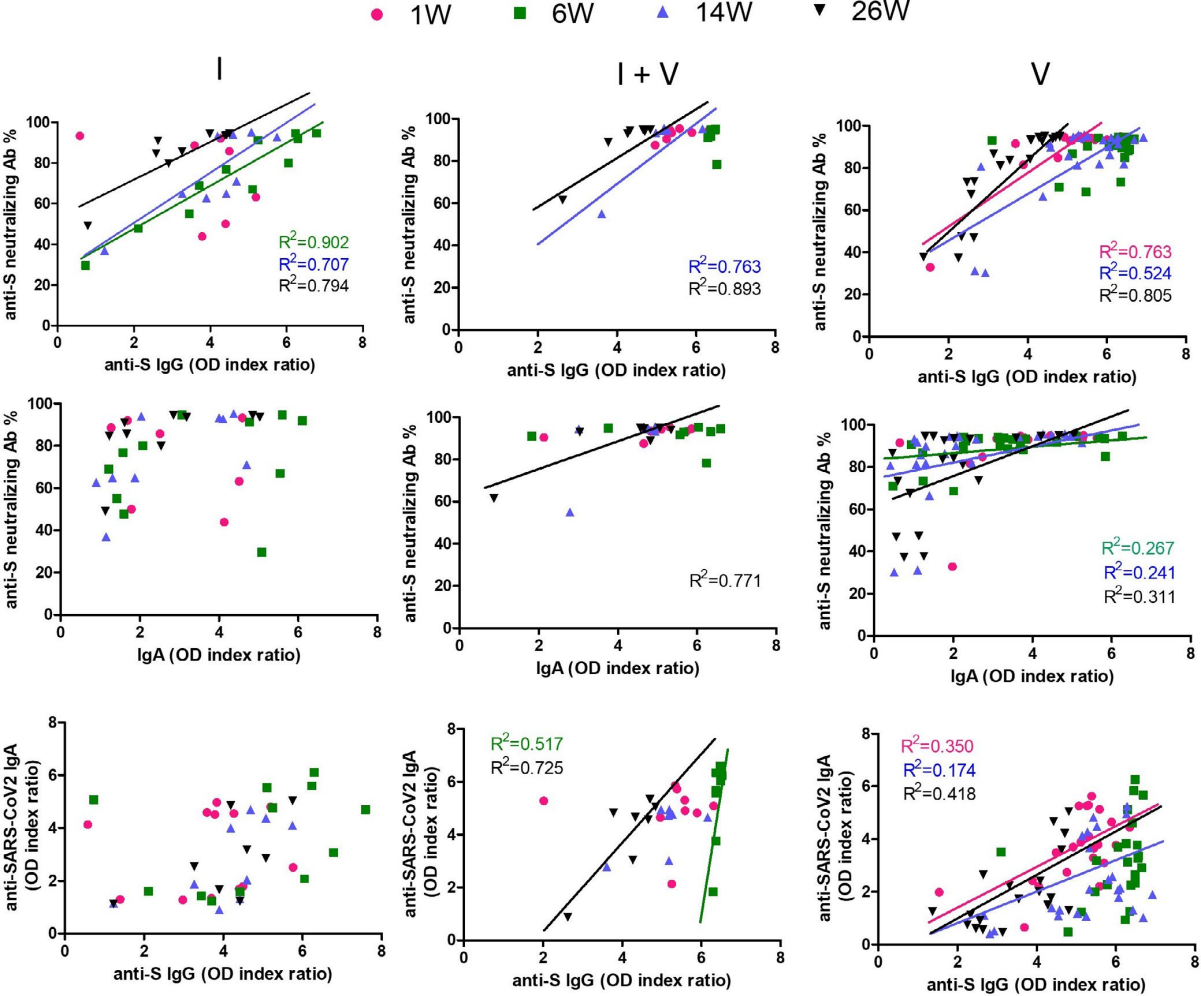
Neutralizing activity positively correlates with the level of anti‐S IgG and IgA antibodies. Correlation of plasma anti‐S IgG, IgA and neutralizing capacity of anti‐S antibodies in different groups of individuals analysed by ELISA. Data were analysed using nonlinear regression and two‐tailed Pearson *r* correlation coefficient. Significant correlations (*p* > 0.05) were presented on graphs via trendlines. Each colour is specific to a time point, according to figure legend

Significant correlations could be observed between the anti‐S IgG response and the inhibition of ACE2 binding through anti‐RBD antibodies in the surrogate neutralization tests. Correlation's analysis showed that individuals with high anti‐S IgG reactivity have higher ACE2 blockade (Figure 4). Similar positive correlations have been observed between neutralizing activity and the level of anti‐S IgA antibodies and between specific IgG and IgA levels in most of the subjects. These observations suggest that anti‐S IgG antibody determination could be valuable in predicting the neutralizing capacity of antibodies in patient samples.

### 3.4 Anti‐SARS‐CoV‐2‐specific cellular immunity

**FIGURE 5:**
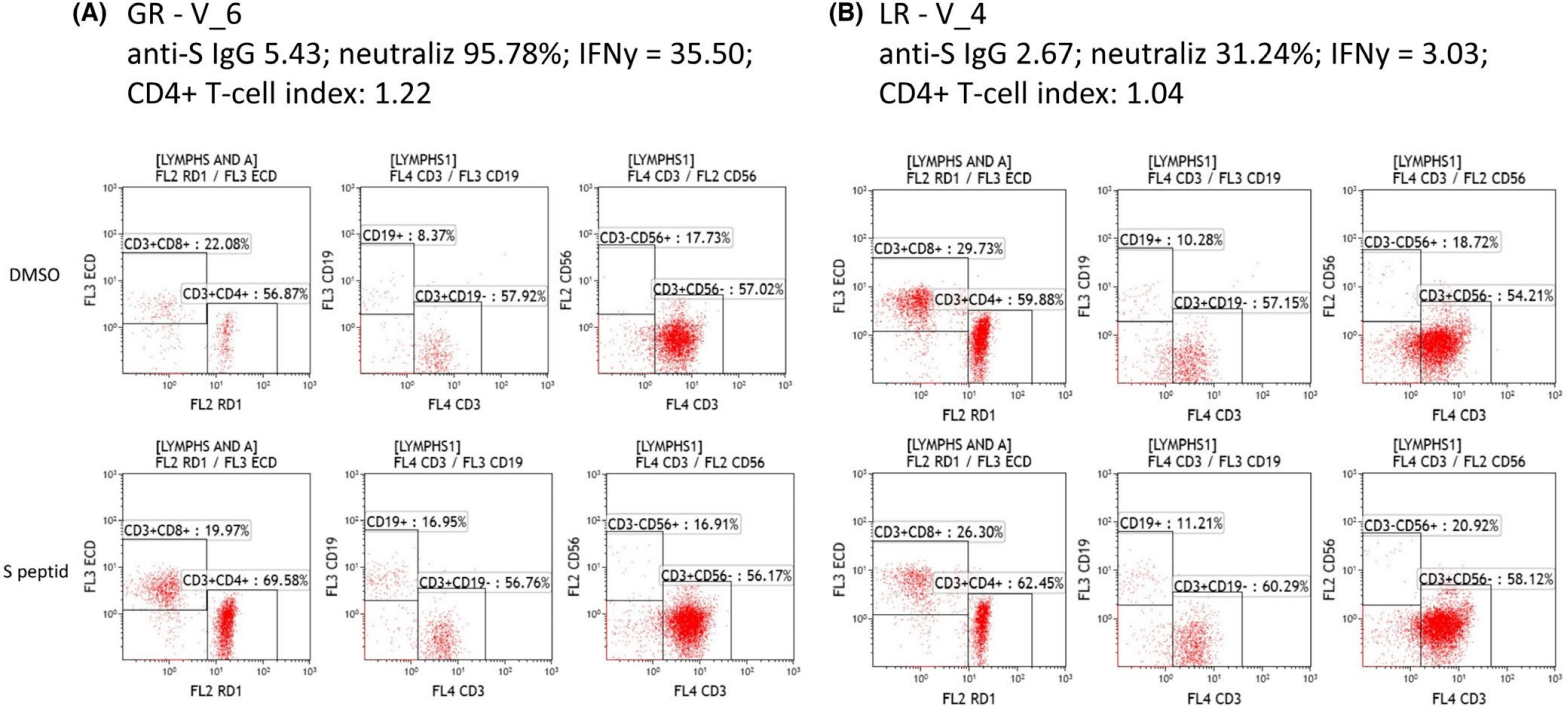
SARS‐CoV‐2‐specific TCD4, TCD8 cells, as well as B and NK cells, were analysed after ex vivo stimulation of PBMCs with PepTivator for 20 h. Individual representative cases showing variation of cellular response specific for SARS‐CoV‐2 vaccinated subjects. (A) A representative sample from a good responder with high Ab titre and neutralizing capacity (GR). (B) A representative sample from a low responder with low Ab titre and neutralizing capacity (LR)

A subset of patients, representing 35.48% (11/31) of V group, 11.11% (1/9) of I + V and 33.33% (4/12) of I group had more pronounced decreasing antibody levels (index ratio 1.1–3) or low neutralizing capacity (30%–40%) during the 6 months follow‐up. They were defined as low responders (LR) and were selected for analysis of the cellular immune response. We compared their cellular immune responses to those in subjects with high antibody ratio (>5.5) or neutralizing capacity (>85%), defined as good responders (GR). We directly assessed capacity of CD4 and CD8 T‐cell subsets to specifically recognize S peptide derived from the ancestral reference sequence (PepTivator SARS‐CoV‐2 Prot_S, Miltenyi Biotec) and B‐cell response (Figure 5). The results were expressed as stimulation index calculated by dividing the measured T CD4^+^, T CD8^+^ and B‐cell subset response by the respective response in the DMSO control. The values indicated a higher CD4^+^ stimulation index with a lower CD8^+^ index for GR compared with LR. This result suggests that SARS‐CoV‐2‐specific CD8^+^ cells are not actively involved in IFN‐γ secretion. There is a significant difference between low and good responders in terms of CD4^+^ (*p* = 0.019), CD8^+^ (*p* = 0.009) T‐cell stimulation index and IFN‐γ secretion (*p* = 0.0007), as well as a trend towards a superior B‐cell reaction in good responders (Table 1).

**TABLE 1:** Comparative analysis of SARS‐CoV‐2 spike‐specific T‐cell and B‐cell responses

Condition	Sample ID	B cells stimulation index	CD4^+^ T cells stimulation index	CD8^+^ T cells stimulation index	anti‐S IgG	Neutralizing capacity %	Neutralizing antibody GMT	IFNɣ (pg/ml)
Good responders (GR) – high Ab titre and neutralizing capacity	V_3	2.36	1.02	0.77	4.58	89.89	64	31.49
	V_6	2.02	1.22	0.95	5.43	95.78	512	35.5
	I + V_2	1.02	1.02	0.95	6.16	95.33	512	20.71
	I + V_3	1.41	1.11	0.76	4.98	93.35	256	45.82
	I + V_5	1.27	1.04	0.95	5.17	94.47	256	53.34
	I + V_6	1.62	1.14	0.9	5	93.5	256	18.52
	I_4	1.46	1.16	0.66	4.19	93.3	128	22.3
	I_3	1.08	1.09	0.8	4.42	80.66	32	15.37
	Mean	1.53	1.10	0.84	4.99	92.04	181.01	30.38
Low responders (LR) – low Ab titre and neutralizing capacity	V_2	1.54	1.05	0.95	2.93	30.25	4	5.46
	V_4	1.09	1.04	0.88	2.67	31.24	8	3.03
	V_5	1.67	0.9	1.42	4.38	66.56	16	4.74
	I + V_1	1.18	1.07	0.92	3.61	55.01	32	7.46
	I_1	0.64	1.03	1.03	3.27	64.94	32	16.35
	I_2	1.06	0.95	1.15	0.73	30.24	16	9.92
	I_5	1.33	1.08	1	1.23	36.94	32	3.07
	I_6	0.82	0.86	1.39	4.42	64.99	16	15.07
	Mean	1.17	1.00	1.09	2.91	47.52	15.99	8.14
*p* Value GR vs LR		9.56E‐02	1.95E‐02	9.77E‐03	1.38E‐03	4.95E‐06	2.97E‐03	7.52E‐04

**FIGURE 6:**
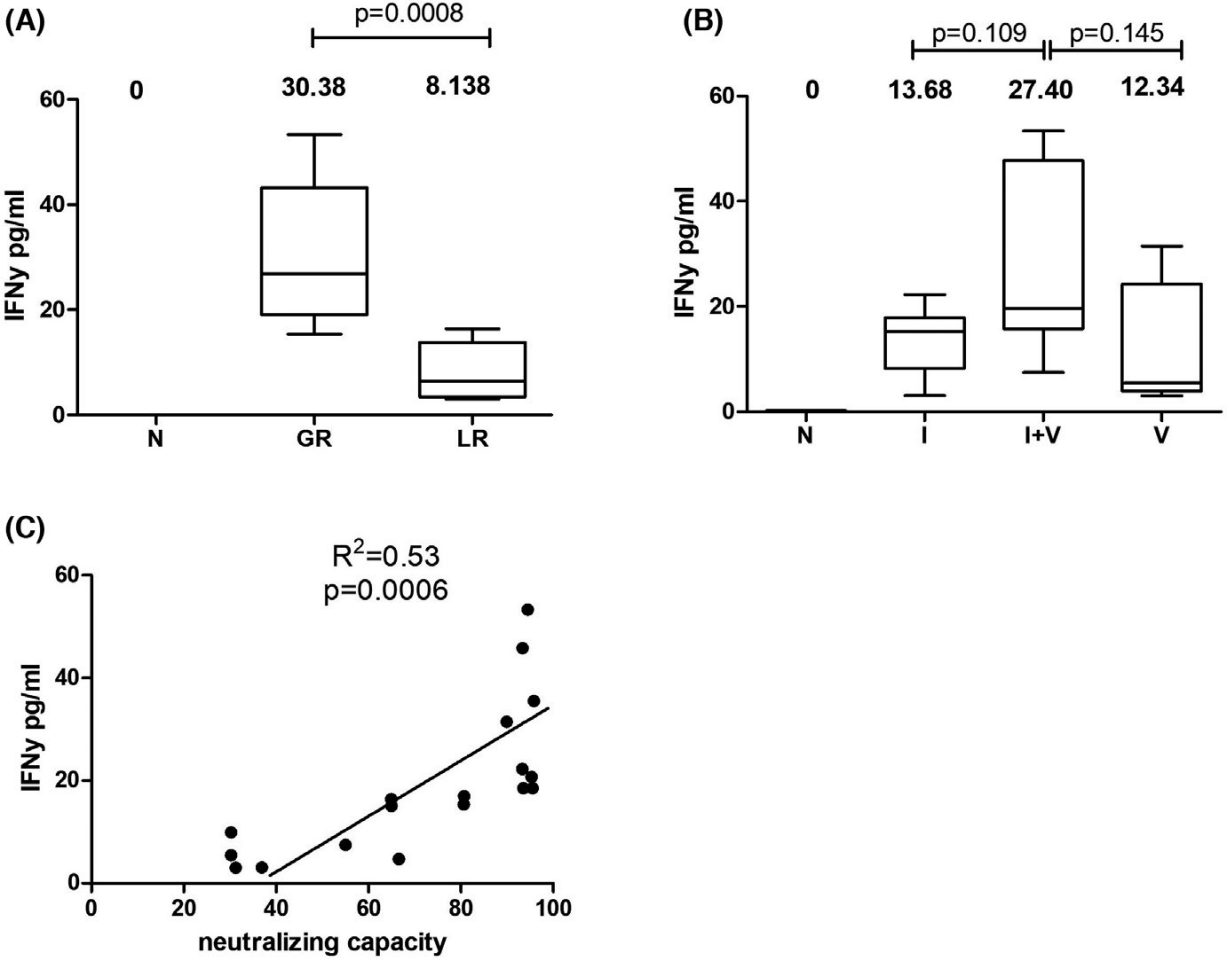
Interferon secretion post–SARS‐CoV‐2‐specific stimulation. (A) Comparative assessment between good responders (GR) with high Ab titre and neutralizing capacity, and low responders (LR) with low Ab titre and neutralizing capacity. (B) Comparative analysis between categories according to infection with or without vaccination. (C) Positive correlation between IFN‐γ secretion and neutralizing capacity

A comparison between the three groups showed that subjects from the I + V category are better IFN‐γ secretors, a fact correlated with a superior T‐cell stimulation index (Figure 6). However, detectable levels of secreted IFN‐**γ** under SARS‐CoV‐2‐specific stimulation was seen in all participant categories, showing that cellular immune memory to SARS‐CoV‐2 develops with large variability driven by the complexity of individual immunity. Subjects with natural infection and those who have been vaccinated without prior infection tend to have a high heterogeneity in the magnitude of SARS‐CoV‐2‐specific cellular immune responses, while those vaccinated after a prior infection have homogenously stronger cellular immune responses.

## 4 DISCUSSION

The key findings of our study on the kinetics of the specific SARS‐CoV‐2 immune response are as follows: (i) at 6 months after vaccination with an mRNA vaccine or after SARS‐CoV‐2 infection, anti‐S IgG antibodies persist and maintain an important neutralizing capacity in the majority of the subjects; (ii) both vaccinated, and prior infected subjects remain protected after 6 months, without any symptomatic or asymptomatic new infections (documented through anti‐NCP IgM antibody testing), although there is a decrease in IgG and IgA anti‐S antibody level over a 6‐month follow‐up; (iii) neutralizing antibody titres in people with prior SARS‐CoV‐2 infection remained constant for more than 250 days after infection with a mean GMT of 46.98 (95% CI: 19.36–114.28) and effectively protected against reinfection; (iv) cellular immune responses to SARS‐CoV‐2 were variable between study participants, but a homogenous strong cellular immune response was present in subjects vaccinated after a prior infection.

Currently, no definitive correlates of protection have been established, although neutralizing antibodies are constantly associated with preventing symptomatic disease. In agreement with our results, in subjects with natural infection, neutralizing antibodies remain still detectable and able to inhibit the in vitro viral replication for periods between 133 and 372 days after an asymptomatic or severe form of infection, respectively.^19^

Recent studies have suggested that neutralizing titres can also act as surrogate markers for vaccine efficacy, being inversely correlated with the risk of COVID‐19 occurrence at least during the first 4 months after a complete vaccination scheme.^20^ A study using orthogonal serology assays showed persistence for at least 6 months of both neutralizing and binding antibodies induced after vaccination with Moderna's mRNA‐1273 vaccine. The neutralizing capacity was maintained against all circulating variants, with the lowest activity and the faster decline against the Beta variant.^21^

In our study, a much higher heterogeneity was observed for the anti‐S IgA antibodies that can serve as a proxy for the mucosal immunity and local protection. Previous reports have shown that in SARS‐CoV‐2‐infected patients anti‐RBD IgA antibodies were produced early during the infection and decreased faster compared to IgG antibodies.^22^ Similar studies on vaccine recipients showed that a high levels of specific SARS‐CoV‐2 Ig A are produced after the first vaccine dose and maintained during the first month post completion of the immunization scheme, regardless of prior SARS‐CoV‐2 infection.^23^ Our results on the declining levels of specific IgA during the first 6 months after an mRNA vaccine and the individual variability might help explain the mildly symptomatic infections occurring overtime in a rather low proportion of vaccinated subjects during intense viral circulation in the community. Nevertheless, it will be worth studying the dynamic and neutralizing capacity of the vaccine‐induced IgA antibody in the mucosa of upper respiratory tract, as these are more potent blockers of the local viral replication. Recent reports showed absence of correlation between early vaccine‐induced serum IgA levels and virus neutralization, but suggested a possible role for non‐neutralizing, Fc binding IgA antibodies in the local immunity.^24^ However, other factors such as the infecting viral variant, the inoculum dose, the type, dose and interval between the vaccine doses, the patients’ comorbidities and degree of immunosuppression may play an essential role in breakthrough infections. Overall, the declining titres of specific IgA and IgG antibodies reflect the normal kinetic of an immune response after natural exposure or vaccination, and therefore, this decrease cannot serve by itself as an indicator for the need of a vaccine booster dose. Substantial heterogeneity exists between the various commercial serological assays used to monitor the dynamic of antibodies post‐infection and post‐vaccination, but there is a wide consensus that virus neutralization tests remain the reference for monitoring the functional humoral immune response.^25^

Yet, even subjects with very low or undetectable levels of antibodies preserve a certain degree of protection. Some studies suggested that significantly lower neutralizing antibody titres are needed for protection after vaccination compared to prior infection (20% of mean convalescent level for protection against detectable infection and 3% for protection against severe infection).^26^ Animal studies have already supported this idea. Relatively low antibody titres, transferred as purified IgG from convalescent animals, were sufficient for protection against experimental SARS‐CoV‐2 challenge in rhesus macaques (Macaca mulatta), even in the absence of cellular and innate immunity.^27^ Ferrets with moderate‐to‐high antibody titres (ie 1:20 to 1:160) obtained after experimental SARS‐CoV‐2 infection were protected during re‐challenge and were unable to transmit the virus to other uninfected animals.^28^

Moreover, the loss of neutralizing antibodies in plasma may be counteracted by the operative memory B cells, as B‐cell neutralizing responses have been detected up to 6 months after SARS‐CoV‐2 infection.^29^ Consistent with these results, our data also identified reactive B cells in peripheral blood in 14 out of 16 cases, with higher stimulation index in good responders compared with low responders in terms of neutralizing capacity or anti‐S IgG antibodies. The stimulation indexes for B cells and CD4 T cells were correlated with the secreted anti‐S IgG antibodies, the neutralizing capacity and the IFN‐γ level. These data are supported by studies demonstrating a correlation between anti‐S antibody titres, and the frequency of S‐specific plasma cells in bone marrow aspirates^30^ and induction of a persistent lymph node germinal centre for B‐cell response after SARS‐CoV‐2 mRNA vaccination allowing a long‐lasting humoral response.^31^ The persistence of virus‐specific memory B cells has been demonstrated at more than 240 days of COVID‐19 symptom onset, even though plasma antibody levels were declining.^32^, ^33^

Additionally, in our study, an important decrease in the anti‐S IgG titres seems to be compensated by higher neutralizing capacity and a robust cellular immune response, demonstrated by a high level of IFN‐γ synthesis by stimulated T cells. Whole‐blood cytokine release assays after stimulation with SARS‐CoV‐2‐specific peptides have been already proposed as an additional diagnostic tool.^34^

Our study also brings essential information regarding SARS‐CoV‐2‐specific T‐cell responses. It is observed that in good responders the CD4^+^ T‐cell index is correlated with a higher secretion of IFN‐γ. This IFN‐γ secretion mainly by TCD4^+^ cells seems to be specific to SARS‐CoV‐2 infection. Several studies have focused on the aspect of the cellular source of IFN‐γ secretion using either scRNA‐seq analysis or flow cytometry. Thus, in‐depth studies were performed to compare the gene expression profiles of SARS‐CoV‐2‐reactive CD8^+^ T‐cell population, with influenza A virus (IAV)‐reactive and respiratory syncytial virus (RSV)‐reactive CD8^+^ T‐cell populations by scRNA‐seq analysis. The results showed that SARS‐CoV‐2‐reactive CD8^+^ T cells had exhibit exhausted phenotypes with type I IFN stimulation and have a decreased capacity to secrete inflammatory cytokines compared to IAV‐reactive or RSV‐reactive CD8^+^ T cells where gene expression profiles were enriched with inflammatory cytokine genes.^35^ Similar results on memory T‐cell responses were also reported by Rha Min‐Seok et al.^36^ who describe that SARS‐CoV‐2‐specific CD8^+^ T cells were functional but proportion of IFN‐γ‐producing cells was significantly lower in COVID‐19 convalescents than those specific to influenza A virus. Moreover, a study performed by Ferreras C. et al. showed that CD4^+^ cells from T central memory and T effector memory subsets, exhibited more IFN‐γ+ cells than CD8^+^ cells with a ratio of 1.17–1.59 (*p* < 0.05).^37^

We observed high heterogeneity in the magnitude of individual cellular immune responses to SARS‐CoV‐2 between study participants. The only ones with a homogenous cellular immune response appear to be those vaccinated after a prior infection, a result sustained by previous observations documenting higher titres of neutralizing antibodies and a broader neutralizing capacity against all circulating variants, as well as persistent robust cellular immune memory responses elicited by a single dose of vaccine in previously infected persons.^38^, ^39^

For unvaccinated individuals with natural infection, it seems that disease severity may contribute to the heterogeneity of cellular immune response, as those asymptomatic or mildly symptomatic had a lower T‐cell stimulation index compared with the ones with moderate forms (results not shown), probably due to lower viral inoculation or lower viral load. Even if the reduced number of analysed samples limits our conclusions, the results align with several studies showing an association between disease severity with a more robust response of virus‐specific T cells. Although there is large variability in the methods used and limited data on the level of cellular immune responses in different categories of subjects,^40^, ^41^ cellular immunity plays an important role in the clearance of viral infection and can prevent the progression of an infection in vaccinated or previously infected individuals. Recently, it has been suggested that in breakthrough infections in vaccinated persons, shedding of infectious virus is limited in duration and occurs less frequently compared to unvaccinated individuals, despite similar viral load detected in RT‐PCR tests.^42^

Studies related to the persistence of the cellular immune response in previously infected individuals showed a half‐life of 3–5 months for SARS‐CoV‐2‐specific CD4^+^ and CD8^+^ T cells, accompanying an increased number of spike‐specific memory B cells at 6 months.^33^ Importantly, T‐cell responses in infected or vaccinated individuals are not significantly affected by mutations found in the SARS‐CoV‐2 variants.^43^

In conclusion, our study shows the persistence of neutralizing antibodies at 6 months after vaccination with an mRNA vaccine or after SARS‐CoV‐2 prior infection, with individual variability associated with specific antibodies’ level and dynamics. Although there is a downward trend in the titre of IgG and especially in IgA anti‐S antibodies, the neutralizing capacity is maintained, and effective cellular immune responses are mounted even in low responders. Nonetheless, there is considerable heterogeneity in both humoral and cellular immunity. Moreover, the decline in antibodies titre did not translate into reduced protection against symptomatic or asymptomatic disease. The strongest cellular immune response was detected in subjects that were vaccinated after prior natural infection, but there were also subjects with good cellular immune response in vaccinated and individuals recovered after natural infection.

We have to acknowledge the limitations of our study due to the rather small number and homogeneous age of the enrolled subjects (mean age 47.3 years), as such the conclusions cannot be generalized to the whole population, with a wider age range, various degrees of immunosuppression and weaker immune responses in older adults. Nevertheless, the study homogeneity, in terms of age and exposure, and complex analysis of antibody, neutralization and cellular immunity profiles increase power of the analysis of the effective anti‐SARS‐CoV‐2 response being informative for the design of further vaccination strategies for healthcare personnel. Additional studies on reinfection in people immunized naturally or by vaccination are needed to answer important questions about the long‐term protection and the level of neutralizing antibodies and/or other immune correlates matching this protection. A key avenue of investigation suggested by our data is the link between cellular immune response and combination of prior infection and vaccination.

## CONFLICT OF INTEREST

The authors declare that the research was conducted in the absence of any commercial or financial relationships that could be construed as a potential conflict of interest.

## AUTHOR CONTRIBUTIONS

**Mihaela Chivu‐Economescu:** Conceptualization (equal); Data curation (equal); Formal analysis (equal); Investigation (lead); Methodology (equal); Writing – original draft (lead); Writing – review & editing (equal). **Coralia Bleotu:** Conceptualization (equal); Data curation (equal); Formal analysis (equal); Investigation (equal); Methodology (equal); Writing – review & editing (equal). **Camelia Grancea:** Data curation (equal); Formal analysis (equal); Investigation (equal); Methodology (equal); Writing – review & editing (equal). **Daniela Chiriac:** Data curation (equal); Formal analysis (equal); Investigation (equal); Methodology (equal); Writing – review & editing (equal). **Anca Botezatu:** Data curation (equal); Formal analysis (equal); Investigation (equal); Methodology (equal); Writing – review & editing (equal). **Iulia V. Iancu:** Data curation (equal); Formal analysis (equal); Investigation (equal); Methodology (equal); Writing – review & editing (equal). **Ioana Pitica:** Data curation (equal); Formal analysis (equal); Investigation (equal); Methodology (equal); Writing – review & editing (equal). **Laura Georgiana Necula:** Data curation (equal); Formal analysis (equal); Investigation (equal); Methodology (equal); Writing – review & editing (equal). **Ana I. Neagu:** Data curation (equal); Formal analysis (equal); Investigation (equal); Methodology (equal); Writing – review & editing (equal). **Lilia Matei:** Data curation (equal); Formal analysis (equal); Investigation (equal); Methodology (equal); Writing – review & editing (equal). **Denisa Dragu:** Data curation (equal); Formal analysis (equal); Investigation (equal); Methodology (equal); Writing – review & editing (equal). **Camelia Sultana:** Data curation (equal); Formal analysis (equal); Investigation (equal); Methodology (equal); Writing – review & editing (equal). **Elena L. Radu:** Data curation (equal); Formal analysis (equal); Investigation (equal); Methodology (equal); Writing – review & editing (equal). **Alina Nastasie:** Data curation (equal); Investigation (equal); Methodology (equal); Writing – review & editing (equal). **Oana Luminita Voicu:** Data curation (equal); Methodology (equal); Writing – review & editing (equal). **Marius Ataman:** Data curation (equal); Investigation (equal); Methodology (equal); Writing – review & editing (equal). **Saviana Nedeianu:** Data curation (equal); Formal analysis (equal); Investigation (equal); Methodology (equal); Writing – review & editing (equal). **Cristina Mambet:** Data curation (equal); Formal analysis (equal); Investigation (equal); Methodology (equal); Writing – review & editing (equal). **Carmen Cristina Diaconu:** Conceptualization (equal); Funding acquisition (equal); Methodology (equal); Project administration (lead); Supervision (equal); Validation (equal); Writing – review & editing (equal). **Simona Ruta:** Conceptualization (equal); Funding acquisition (equal); Supervision (equal); Validation (equal); Writing – original draft (equal); Writing – review & editing (equal).

## Data Availability

The data that support the findings of this study are available from the corresponding author upon reasonable request.
